# 1,4-Dichloro-2,3-bis­(chloro­meth­yl)butane

**DOI:** 10.1107/S1600536812048398

**Published:** 2012-11-30

**Authors:** David B. Cordes, Kati M. Aitken, R. Alan Aitken

**Affiliations:** a EaStCHEM School of Chemistry, University of St Andrews, Fife KY16 9ST, Scotland

## Abstract

The title compound, C_6_H_10_Cl_4_, adopts a geometric arrangement with two C—Cl bonds anti­periplanar to C—H bonds and the other two anti­periplanar to C—C bonds. While minimising steric replusion, this arrangement still gives rise to some intramolecular C—H⋯Cl contacts. In the crystal, mol­ecules are connected into a three-dimensional architecture *via* further C—H⋯Cl contacts.

## Related literature

The title compound was previously prepared by Weinges & Spänig (1968 ▶). For related structures of polychlorinated acylic alkanes, see: Frenzen *et al.* (1999 ▶); Frenzen & Coelhan (1998 ▶); Bart *et al.* (1979 ▶, 1980 ▶); Karapetyan *et al.* (2008 ▶); Kabalka *et al.* (2005 ▶); Podsiadło & Katrusiak (2006 ▶); Klaeboe *et al.* (1986 ▶).
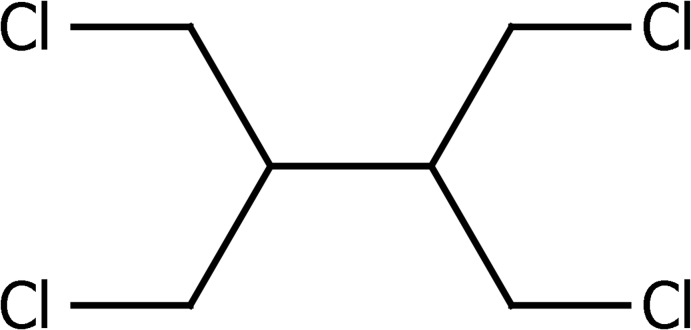


## Experimental

### 

#### Crystal data

C_6_H_10_Cl_4_
*M* *_r_* = 223.94
Orthorhombic, 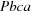

*a* = 8.998 (3) Å
*b* = 8.400 (3) Å
*c* = 24.643 (7) Å
*V* = 1862.6 (10) Å^3^
*Z* = 8
Mo *K*α radiation
μ = 1.20 mm^−1^
*T* = 93 K
0.25 × 0.25 × 0.10 mm

#### Data collection

Rigaku Mercury diffractometer
Absorption correction: multi-scan (*CrystalClear*; Rigaku, 2010 ▶) *T*_min_ = 0.746, *T*_max_ = 1.000
8405 measured reflections
1658 independent reflections
1553 reflections with *I* > 2σ(*I*)
*R*_int_ = 0.050

#### Refinement

*R*[*F* ^2^ > 2σ(*F* ^2^)] = 0.030
*wR*(*F* ^2^) = 0.075
*S* = 1.12
1658 reflections
91 parameters
H-atom parameters constrained
Δρ_max_ = 0.29 e Å^−3^
Δρ_min_ = −0.26 e Å^−3^

### 

Data collection: *CrystalClear* (Rigaku, 2010 ▶); cell refinement: *CrystalClear*; data reduction: *CrystalClear*; program(s) used to solve structure: *SIR2004* (Burla *et al.*, 2005 ▶); program(s) used to refine structure: *SHELXTL* (Sheldrick, 2008 ▶); molecular graphics: *SHELXTL*; software used to prepare material for publication: *SHELXTL* and *publCIF* (Westrip, 2010 ▶).

## Supplementary Material

Crystal structure: contains datablock(s) global, I. DOI: 10.1107/S1600536812048398/tk5174sup1.cif

Structure factors: contains datablock(s) I. DOI: 10.1107/S1600536812048398/tk5174Isup2.hkl

Supplementary material file. DOI: 10.1107/S1600536812048398/tk5174Isup3.cml

Additional supplementary materials:  crystallographic information; 3D view; checkCIF report

## Figures and Tables

**Table 1:** Hydrogen-bond geometry (Å, °)

*D*—H⋯*A*	*D*—H	H⋯*A*	*D*⋯*A*	*D*—H⋯*A*
C1—H1*B*⋯Cl3	0.99	2.76	3.2097 (19)	108
C4—H4*B*⋯Cl4	0.99	2.80	3.2445 (19)	108
C5—H5*B*⋯Cl1	0.99	2.74	3.2069 (19)	109
C6—H6*B*⋯Cl2	0.99	2.72	3.1940 (18)	110
C2—H2⋯Cl3^i^	1.00	2.93	3.8599 (19)	155
C3—H3⋯Cl2^ii^	1.00	2.86	3.8092 (19)	160
C4—H4*B*⋯Cl3^i^	0.99	2.92	3.657 (2)	132
C5—H5*A*⋯Cl2^iii^	0.99	2.90	3.6951 (19)	138
C6—H6*A*⋯Cl1^iv^	0.99	2.84	3.655 (2)	140

Symmetry codes: (i) 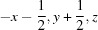; (ii) 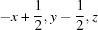; (iii) 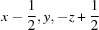; (iv) 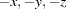.

**Fig. 1.**
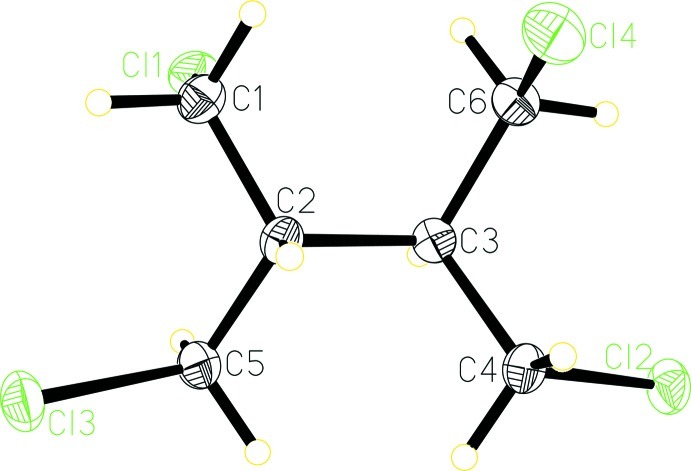
The molecular structure of the title compound with displacement ellipsoids drawn at the 50% probability level.

## supplementary crystallographic information

### Comment

The title compound shows a mixture of geometric arrangements of the C—Cl bonds, with two of them antiperiplanar to C—C bonds [Cl3—C5—C2—C3: 166.68 (11)°, Cl2—C4—C3—C2: 166.96 (11)°], and the other two antiperiplanar to C—H bonds [Cl1—C1—C2—H2: 178.6°, Cl4—C6—C3—H3: 175.9°]. This pattern of differing geometric arrangements has also been seen in related polychlorinated acylic alkanes (Frenzen *et al.*, 1999; Frenzen & Coelhan, 1998; Bart *et al.*, 1979, 1980; Karapetyan *et al.*, 2008; Kabalka *et al.*, 2005; Podsiadło & Katrusiak, 2006; Klaeboe *et al.*, 1986), due to the necessity of minimizing steric repulsion in such extended structures. The arrangement of the C—Cl bonds gives rise to intramolecular C—H···Cl contacts for all four chlorines, at distances ranging from 2.72 to 2.80 Å. In addition, three of the four chlorine atoms also make intermolecular C—H···Cl contacts to adjacent molecules, at distances between 2.84 and 2.93 Å, resulting in the formation of a weakly interacting three-dimensional array.

### Experimental

The title compound was prepared by the method of Weinges and Spänig (1968). Crystals suitable for X-ray structure determination were obtained by sublimation at room temperature and ambient pressure.

### Refinement

Carbon-bound H atoms were included in calculated positions (C—H distances are 1.00 Å for methine H atoms and 0.99 Å for methylene H atoms) and refined as riding atoms with *U*_iso_(H) = 1.2 *U*_eq_(parent atom).

### Crystal data

**Table:**

C_6_H_10_Cl_4_	*F*(000) = 912
*M**_r_* = 223.94	*D*_x_ = 1.597 Mg m^−^^3^
Orthorhombic, *P**b**c**a*	Mo *K*α radiation, λ = 0.71073 Å
Hall symbol: -P 2ac 2ab	Cell parameters from 5355 reflections
*a* = 8.998 (3) Å	θ = 1.7–28.6°
*b* = 8.400 (3) Å	µ = 1.20 mm^−^^1^
*c* = 24.643 (7) Å	*T* = 93 K
*V* = 1862.6 (10) Å^3^	Prism, colourless
*Z* = 8	0.25 × 0.25 × 0.10 mm

### Data collection

**Table:**

Rigaku Mercury diffractometer	1658 independent reflections
Radiation source: rotating anode	1553 reflections with *I* > 2σ(*I*)
Confocal monochromator	*R*_int_ = 0.050
Detector resolution: 14.7059 pixels mm^-1^	θ_max_ = 25.4°, θ_min_ = 2.8°
ω and φ scans	*h* = −10→10
Absorption correction: multi-scan (*CrystalClear*; Rigaku, 2010)	*k* = −9→10
*T*_min_ = 0.746, *T*_max_ = 1.000	*l* = −25→29
8405 measured reflections	

### Refinement

**Table:**

Refinement on *F*^2^	Primary atom site location: structure-invariant direct methods
Least-squares matrix: full	Secondary atom site location: difference Fourier map
*R*[*F*^2^ > 2σ(*F*^2^)] = 0.030	Hydrogen site location: inferred from neighbouring sites
*wR*(*F*^2^) = 0.075	H-atom parameters constrained
*S* = 1.12	*w* = 1/[σ^2^(*F*_o_^2^) + (0.0315*P*)^2^ + 0.758*P*] where *P* = (*F*_o_^2^ + 2*F*_c_^2^)/3
1658 reflections	(Δ/σ)_max_ < 0.001
91 parameters	Δρ_max_ = 0.29 e Å^−^^3^
0 restraints	Δρ_min_ = −0.26 e Å^−^^3^

### Special details

**Table:**

Geometry. All e.s.d.'s (except the e.s.d. in the dihedral angle between two l.s. planes) are estimated using the full covariance matrix. The cell e.s.d.'s are taken into account individually in the estimation of e.s.d.'s in distances, angles and torsion angles; correlations between e.s.d.'s in cell parameters are only used when they are defined by crystal symmetry. An approximate (isotropic) treatment of cell e.s.d.'s is used for estimating e.s.d.'s involving l.s. planes.
Refinement. Refinement of *F*^2^ against ALL reflections. The weighted *R*-factor *wR* and goodness of fit *S* are based on *F*^2^, conventional *R*-factors *R* are based on *F*, with *F* set to zero for negative *F*^2^. The threshold expression of *F*^2^ > σ(*F*^2^) is used only for calculating *R*-factors(gt) *etc*. and is not relevant to the choice of reflections for refinement. *R*-factors based on *F*^2^ are statistically about twice as large as those based on *F*, and *R*- factors based on ALL data will be even larger.

### Fractional atomic coordinates and isotropic or equivalent isotropic displacement parameters (Å^2^)

**Table:**

	*x*	*y*	*z*	*U*_iso_*/*U*_eq_	
Cl1	−0.07005 (5)	−0.12175 (5)	0.063173 (16)	0.02498 (15)	
Cl2	0.32971 (5)	0.25183 (6)	0.190640 (17)	0.02602 (15)	
Cl3	−0.33730 (5)	0.06538 (6)	0.184618 (18)	0.02712 (15)	
Cl4	0.08315 (5)	0.40738 (5)	0.056565 (18)	0.02950 (15)	
C1	−0.1427 (2)	0.0759 (2)	0.07550 (7)	0.0210 (4)	
H1A	−0.1140	0.1468	0.0452	0.025*	
H1B	−0.2526	0.0714	0.0768	0.025*	
C2	−0.08431 (17)	0.14452 (19)	0.12866 (6)	0.0177 (4)	
H2	−0.1265	0.2541	0.1324	0.021*	
C3	0.08737 (17)	0.16035 (19)	0.13078 (6)	0.0174 (4)	
H3	0.1284	0.0520	0.1383	0.021*	
C4	0.13327 (19)	0.2676 (2)	0.17793 (6)	0.0215 (4)	
H4A	0.0777	0.2367	0.2110	0.026*	
H4B	0.1079	0.3795	0.1693	0.026*	
C5	−0.13917 (19)	0.0475 (2)	0.17705 (7)	0.0216 (4)	
H5A	−0.0896	0.0855	0.2105	0.026*	
H5B	−0.1126	−0.0658	0.1718	0.026*	
C6	0.15708 (18)	0.2177 (2)	0.07798 (7)	0.0207 (4)	
H6A	0.1388	0.1378	0.0492	0.025*	
H6B	0.2659	0.2273	0.0829	0.025*	

### Atomic displacement parameters (Å^2^)

**Table:**

	*U*^11^	*U*^22^	*U*^33^	*U*^12^	*U*^13^	*U*^23^
Cl1	0.0309 (3)	0.0212 (3)	0.0228 (2)	−0.00077 (18)	0.00016 (16)	−0.00473 (16)
Cl2	0.0180 (3)	0.0320 (3)	0.0281 (3)	−0.00443 (16)	−0.00639 (15)	0.00751 (18)
Cl3	0.0168 (3)	0.0315 (3)	0.0331 (3)	−0.00376 (17)	0.00498 (16)	−0.00645 (18)
Cl4	0.0355 (3)	0.0231 (3)	0.0299 (3)	−0.00145 (18)	−0.00172 (17)	0.00903 (18)
C1	0.0210 (9)	0.0203 (8)	0.0215 (8)	0.0006 (7)	−0.0031 (7)	0.0003 (7)
C2	0.0157 (9)	0.0173 (8)	0.0201 (8)	0.0012 (6)	−0.0019 (6)	−0.0006 (7)
C3	0.0167 (9)	0.0167 (8)	0.0188 (8)	0.0017 (6)	−0.0003 (6)	0.0010 (6)
C4	0.0141 (8)	0.0286 (9)	0.0219 (8)	−0.0014 (7)	−0.0021 (6)	−0.0004 (7)
C5	0.0157 (8)	0.0275 (9)	0.0217 (8)	0.0002 (7)	0.0012 (6)	−0.0022 (7)
C6	0.0210 (10)	0.0196 (8)	0.0213 (8)	0.0012 (7)	0.0010 (6)	0.0013 (7)

### Geometric parameters (Å, º)

**Table:**

Cl1—C1	1.8103 (18)	C3—C6	1.523 (2)
Cl2—C4	1.7999 (18)	C3—C4	1.528 (2)
Cl3—C5	1.7987 (18)	C3—H3	1.0000
Cl4—C6	1.8054 (18)	C4—H4A	0.9900
C1—C2	1.525 (2)	C4—H4B	0.9900
C1—H1A	0.9900	C5—H5A	0.9900
C1—H1B	0.9900	C5—H5B	0.9900
C2—C5	1.526 (2)	C6—H6A	0.9900
C2—C3	1.551 (2)	C6—H6B	0.9900
C2—H2	1.0000		
			
C2—C1—Cl1	111.49 (11)	C3—C4—Cl2	110.75 (12)
C2—C1—H1A	109.3	C3—C4—H4A	109.5
Cl1—C1—H1A	109.3	Cl2—C4—H4A	109.5
C2—C1—H1B	109.3	C3—C4—H4B	109.5
Cl1—C1—H1B	109.3	Cl2—C4—H4B	109.5
H1A—C1—H1B	108.0	H4A—C4—H4B	108.1
C1—C2—C5	110.98 (14)	C2—C5—Cl3	110.91 (12)
C1—C2—C3	113.87 (13)	C2—C5—H5A	109.5
C5—C2—C3	109.97 (13)	Cl3—C5—H5A	109.5
C1—C2—H2	107.2	C2—C5—H5B	109.5
C5—C2—H2	107.2	Cl3—C5—H5B	109.5
C3—C2—H2	107.2	H5A—C5—H5B	108.0
C6—C3—C4	110.60 (14)	C3—C6—Cl4	112.17 (11)
C6—C3—C2	114.11 (13)	C3—C6—H6A	109.2
C4—C3—C2	110.21 (13)	Cl4—C6—H6A	109.2
C6—C3—H3	107.2	C3—C6—H6B	109.2
C4—C3—H3	107.2	Cl4—C6—H6B	109.2
C2—C3—H3	107.2	H6A—C6—H6B	107.9
			
Cl1—C1—C2—C5	−64.59 (16)	C2—C3—C4—Cl2	166.96 (11)
Cl1—C1—C2—C3	60.14 (17)	C1—C2—C5—Cl3	−66.41 (15)
C1—C2—C3—C6	40.2 (2)	C3—C2—C5—Cl3	166.68 (11)
C5—C2—C3—C6	165.43 (14)	C4—C3—C6—Cl4	−67.52 (15)
C1—C2—C3—C4	165.31 (13)	C2—C3—C6—Cl4	57.41 (17)
C5—C2—C3—C4	−69.43 (18)	Cl1—C1—C2—H2	178.6
C6—C3—C4—Cl2	−65.92 (15)	Cl4—C6—C3—H3	175.9

### Hydrogen-bond geometry (Å, º)

**Table:**

*D*—H···*A*	*D*—H	H···*A*	*D*···*A*	*D*—H···*A*
C1—H1*B*···Cl3	0.99	2.76	3.2097 (19)	108
C4—H4*B*···Cl4	0.99	2.80	3.2445 (19)	108
C5—H5*B*···Cl1	0.99	2.74	3.2069 (19)	109
C6—H6*B*···Cl2	0.99	2.72	3.1940 (18)	110
C2—H2···Cl3^i^	1.00	2.93	3.8599 (19)	155
C3—H3···Cl2^ii^	1.00	2.86	3.8092 (19)	160
C4—H4*B*···Cl3^i^	0.99	2.92	3.657 (2)	132
C5—H5*A*···Cl2^iii^	0.99	2.90	3.6951 (19)	138
C6—H6*A*···Cl1^iv^	0.99	2.84	3.655 (2)	140

Symmetry codes: (i) −*x*−1/2, *y*+1/2, *z*; (ii) −*x*+1/2, *y*−1/2, *z*; (iii) *x*−1/2, *y*, −*z*+1/2; (iv) −*x*, −*y*, −*z*.
